# Adaptive treatment margins to reduce organs at risk dose in patients with no or minimal anatomical changes in radiotherapy of non-small cell lung cancer

**DOI:** 10.1016/j.phro.2025.100699

**Published:** 2025-01-20

**Authors:** Djoya Hattu, Daisy Emans, Janine Bouten, Richard Canters, Judith van Loon, Dirk De Ruysscher

**Affiliations:** Department of Radiation Oncology (MAASTRO), GROW-School for Oncology and Reproduction, Maastricht University Medical Center, Maastricht, the Netherlands

**Keywords:** Non-small cell lung cancer, reduced PTV margins, Adaptive radiotherapy, Plan of the day, Library of plans, IGRT

## Abstract

**Background and purpose:**

In non-small cell lung cancer (NSCLC) a significant portion of the planning target volume (PTV) margin accommodates for anatomical changes during treatment. Patients with no or minimal anatomical changes might therefore benefit from a reduced PTV margin, resulting in lower organ at risk (OAR) doses. We evaluated a plan of the day approach using different PTV margins to quantify its effect on OAR and clinical target volume (CTV) dose.

**Materials and methods:**

Twenty NSCLC patients were included in this retrospective study. CBCTs of all fractions were evaluated using an image-guided radiotherapy (IGRT) protocol to classify fractions into two groups: no or minimal anatomical changes to which reduced PTV margin plans (5 or 2 mm) were assigned, or with anatomical changes that received the reference treatment plan (8 mm PTV margin). OAR doses were investigated and CTV coverage was evaluated using CBCT dose recalculations.

**Results:**

All plans showed decreased OAR dose when the PTV margin was reduced from 8 mm to 5 mm or 2 mm. The IGRT protocol selected 254/600 fractions in 19/20 patients, that could be treated with a smaller margin. CTV V_95%_ remained ≥95% in 94% of the 5 mm plans and 87% of the 2 mm plans, compared to 98% of the reference 8 mm plans.

**Conclusion:**

The IGRT protocol could identify fractions with no or minimal anatomical changes allowing a plan of the day approach to reduce PTV margins. Target coverage remained adequate in the majority of patients, while reducing OAR doses.

## Highlights

• Coverage was adequate in 94% of adapted fractions with minimal anatomical changes.
• 49% (294/600) of treatment fractions were eligible for adaptive radiotherapy.
• A plan of the day approach enables target margin reduction in lung cancer patients.

## 1 Introduction

Inoperable stage III non-small cell lung (NSCLC) cancer is predominantly treated with chemo-radiotherapy [1]. The tumor is treated with a specified radiation dose while limiting dose to the surrounding organs at risk (OAR). During radiotherapy delivery, various systematic and random errors can occur that need to be considered, such as patient setup, motion, delineation, and anatomical variation. These errors are taken into account using a planning target volume (PTV) margin to ensure good target coverage of the clinical target volume (CTV) [2], [3], [4]. For lung cancer, a large proportion of the margin is due to the probability of anatomical changes occurring during treatment, potentially causing a shift of the tumor and deviation from the planned dose distribution.

Anatomical changes are very common in lung cancer patients and different types of changes occur during treatment, e.g., change of tumor volume, change of atelectasis, pleural effusion or large shifts of the tumor and/or the OAR [5], [6]. Daily online cone-beam computer tomography (CBCT) for position verification makes it possible to visually evaluate the anatomy and positioning of the patient before treatment [7], [8]. Currently, this workflow is used to correctly position the patient and to determine if the patients’ anatomy is within predefined boundaries. The focus of these protocols is to identify patients that might require a plan adaptation, i.e. adaptive radiotherapy (ART) [9], [10], [11], [12]. However, these workflows could also be used to identify patients that have no or limited anatomical changes, who might benefit from reduced margins without compromising tumor coverage. In other tumor sites, similar plan of the day approaches are already implemented to cover anatomical variation, e.g., in bladder cancer [13], [14], [15], [16], rectal cancer [17], [18], and cervical cancer [19], [20], [21].

The aim of this study was to evaluate the effect of reduced PTV margins on OAR dose and CTV coverage in NSCLC patients. Additionally, we combined an image-guided radiotherapy (IGRT) protocol for ART with a plan of the day approach using different PTV margins, to select eligible fractions with no or minor anatomical changes for margin reduction, and investigated its dose effects.

## 2 Materials and methods

In order to avoid selection bias as much as possible, 20 consecutive stage III NSCLC patients treated with concurrent chemo-radiation therapy in 2019 were selected for this retrospective study. The radiation dose was 60 Gy in 30 daily fractions in 6 weeks. Planning computer tomography (CT) and daily CBCT images (Varian Truebeam OBI, Varian Medical Systems, Siemens Healthineers, Erlangen, Germany) of all treatment fractions were available for evaluation. See Table 1 for patient characteristics.

**Table 1:** Patient and treatment characteristics. GTVp: Gross Tumor Volume of primary tumor, CTVp: Clinical Target Volume of primary tumor, NSCLC: non-small cell lung cancer, NOS: not otherwise specified, IQR: interquartile range.

			**Count (n)**	**%**
	Clinical Stage	IIIa	6	30
		IIIb	14	70
	Clinical Tumor stage	1	1	5
		2	2	10
		3	5	25
		4	12	60
	Clinical Nodal stage	1	2	10
		2	14	70
		3	4	20
	Tumor location	Left lobe	9	45
		Right lobe	11	55
		Upper lobe	11	55
		Lower lobe	9	45
	Tumor Type	Squamous Cell	7	35
		Adeno	8	40
		NSCLC NOS	5	25
	Gender	Female	7	35
		Male	13	65
			**Median**	**IQR**
	GTVp volume (cm^3^)		85	62–226
	CTVp volume (cm^3^)		174	141–384
	Age (y)		66	61–75

**Fig. 1:**
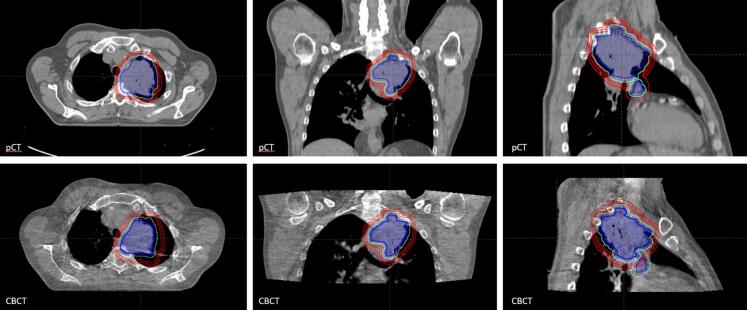
Transverse, coronal and sagittal view of the target volumes with the different margins for the primary tumor on the planning CT (top) and CBCT of a fraction (bottom). The GTV and CTV are adjusted to meet the anatomy of the CBCT, the PTVs are copied rigidly from the planning CT. Red: PTV of primary tumor with different margins (8 mm, 5 mm, 2 mm). Green: PTV of the nodes (5 mm margin). Light blue: CTV. Dark blue: GTV. pCT: planning CT, CBCT: cone-beam CT.

To investigate the effect of smaller margins on the OAR dose, treatment plans were created for different PTV margins. For each patient the clinical target volume of the primary tumor (CTVp) was expanded with an 8 mm margin (reference margin). In three patients this PTV was further expanded with 1 or 2 mm in the direction of tumor motion, because the tumor motion was larger than 5 mm. Additionally, two smaller PTVs were created from the CTVp, one with a 3 mm reduction to a margin of 5 mm, and one with a 6 mm reduction to a margin of 2 mm, resulting in three PTVp structures for each patient (Fig. 1). The margin of the nodes (PTVn) remained unchanged and was a 5 mm expansion of the CTVn.

Treatment plans were made in Eclipse v16 (Varian Medical Systems, Siemens Healthineers, Erlangen, Germany) and consisted of two 180-degree VMAT beams. First, the reference treatment plan with 8 mm margins was created (Plan_8). The plans with smaller margins were created by copying the 8 mm plan with subsequent further optimization for the smaller PTVs. The dosimetrist tried to further lower the dose to the heart, lungs and esophagus in Plan_5 and Plan_2.

OAR were evaluated on the planning CT by extracting dose-volume histogram parameters for all three plans and the effect of the reduced margins on the dose to the OARs was calculated for a best-case scenario, i.e. all fractions with reduced margins (Plan_5 or Plan_2), to determine the maximum benefit. Since it is not known in advance which patients develop anatomical changes during treatment, reducing the PTV margin for the entire population is not without consequences, i.e. some patients will have reduced target coverage. This could lead to more plan adaptations, and thus a higher workload in the clinic. Therefore, an IGRT protocol was used to select patients with no or minimal anatomical changes [12]. Details of this IGRT protocol are repeated in Supplementary Fig. S1 and S upplementary Table S1. In addition to the best-case scenarios, combination scenarios were defined. The CBCT of every fraction was retrospectively evaluated and classified into two groups, i.e. no or minimal anatomical changes (code green), or anatomical changes (code orange). This classification determined which treatment plan was assigned to each fraction, i.e. the plan with reduced margins (code green) or the reference plan (code orange). The two plan doses were scaled to their assigned number of fractions and summed, combining *n* fractions of the reduced margin plan with *m* fractions (*m* = 30 − *n*) of the reference plan, to Dose A (Plan_8 with Plan_5), or Dose B (Plan_8 with Plan_2). Dose differences were calculated between the current treatment strategy (all fractions Plan_8) and the combined scenarios (Dose A, or Dose B). Statistical significance was tested using a paired T-test (IBM SPSS Statistics for Windows, version 29, IBM Corp., Armonk, NY, USA) and a p-value of < 0.05 was considered statistically significant. To correct for multiple testing, the p-values were also tested against a Bonferroni corrected alpha level (p < 0.001, m = 52 hypotheses).

**Fig. 2:**
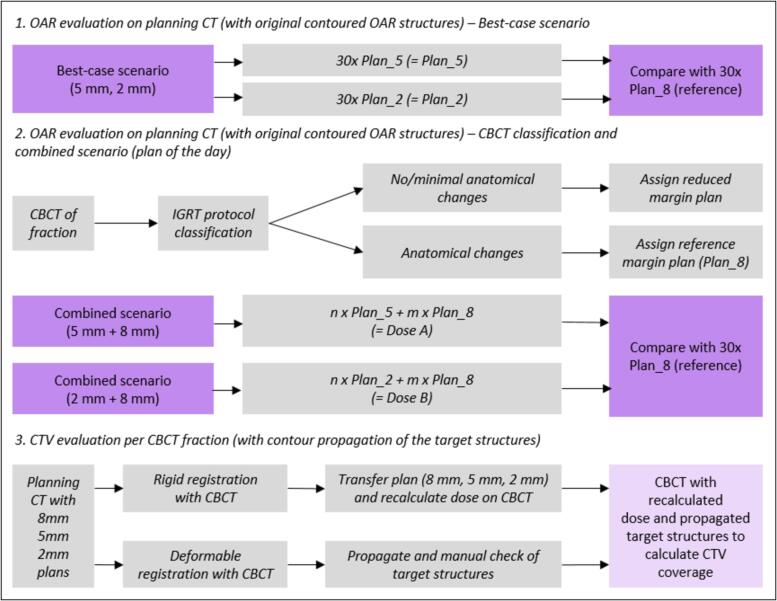
Schematic overview of the study design illustrating the different evaluations. The OAR evaluations were performed on the planning CT with the original OAR contours, the CTV evaluation was performed on the daily CBCT with CTV contour propagation. Purple boxes indicate doses and OAR structures on the planning CT, pink boxes indicate doses and propagated CTV structures on the CBCT. CBCT: Cone-beam CT, IGRT: Image-guided radiotherapy, OAR: organs at risk, CTV: Clinical target volume.

To investigate the effect of different PTV margins on the CTVp coverage, a dose recalculation of each of the three plans (Plan_8, Plan_5, and Plan_2) was performed on every CBCT (CT HU-ED table) [22]. GTV structures were propagated from the planning CT to all CBCTs using a hybrid deformable image registration between the planning CT and each CBCT (RayStation 11B, RaySearch Labs, Stockholm, Sweden). The GTV was visually evaluated by a single observer on all CBCTs in chronological order and adjusted if necessary using a predefined setting (window-width −1000 and window-level 200). The CTVp was regenerated from the GTV. Treatment plans were recalculated on the CBCTs using the online rigid registration between the planning CT and CBCT used during treatment, and the V_95%_ coverage of the CTVp was evaluated. To account for uncertainties such as intrafraction motion and CBCT target delineation, additionally the coverage of a 1 mm expanded CTVp was evaluated. A schematic overview of the study is given in Fig. 2.

## 3 Results

Table 2A depicts the mean doses for all OARs on the planning CT for the two best-case scenarios (Plan_5 and Plan_02) and the reference plan (Plan_8). A reduction of 3 mm led to a decrease of almost all OAR doses. The increase in maximum dose to the body and mediastinal envelope were negligible. The mean dose of the heart decreased with 0.4 Gy, the esophagus with 1.5 Gy, and the lungs with 0.8 Gy. The 2 mm PTV margin plans further decreased the doses in the OARs and showed slightly higher but still negligible increases in the maximum dose of the body an mediastinal envelope. The mean dose to the heart decreased with 0.7 Gy, the esophagus with 2.5 Gy, and the lungs with 1.4 Gy.

**Table 2:** OAR dose evaluation on the planning CT. Part A: Results based on 30 fractions for each of the three PTV margin plans (Plan_8, Plan_5, and Plan_2). Mean and standard deviation of all OAR and mean differences and standard deviation between the two reduced margin plans (Plan_5 and Plan_2) with the reference plan (Plan_8). Part B: Results based on a combination of the reference plan (Plan_8) and the reduced margin plans (Plan_5 or Plan_2), depending on the CBCT classifications. Plan_5 or Plan_2 was assigned when no or minimal anatomical changes were identified. Dose A combined the 8 mm and 5 mm PTV margin plans. Dose B combined the 8 mm and 2 mm PTV margin plans. Asterisks indicate statistical significance (p < 0.05), daggers indicate statistical significance after Bonferroni correction (p < 0.001, m = 52). OAR: Organ at risk, PTV: Planning target volume, GTV: Gross tumor volume.

**A. Best-case scenario**	Plan_8 (Reference)	Plan_5	Plan_2	Difference Plan_5 − Plan_8	P-value	Difference Plan_2 − Plan_8	P-value
Body D_mean_ (Gy)	7.0 ± 2.4	6.5 ± 2.2	6.2 ± 2.1	−0.5 ± 0.2	<0.001†	−0.8 ± 0.4	<0.001†
Heart D_mean_ (Gy)	5.8 ± 3.7	5.4 ± 3.5	5.1 ± 3.4	−0.4 ± 0.4	<0.001†	−0.7 ± 0.7	<0.001†
Esophagus D_mean_ (Gy)	18.4 ± 9.4	16.9 ± 9.0	15.9 ± 8.7	−1.5 ± 1.2	<0.001†	−2.5 ± 2.2	<0.001†
Lungs-GTV D_mean_ (Gy)	12.9 ± 4.2	12.1 ± 4.1	11.5 ± 4.0	−0.8 ± 0.3	<0.001†	−1.4 ± 0.6	<0.001†
Lungs-GTV V_5Gy_ (%)	51.2 ± 18.6	48.9 ± 18.8	46.8 ± 18.5	−2.3 ± 2.7	0.001*	−4.4 ± 3.7	<0.001†
Lung Ipsilateral V_5Gy_ (%)	63.9 ± 19.5	62.6 ± 19.8	61.4 ± 20.0	−1.3 ± 1.1	<0.001†	−2.5 ± 2.0	<0.001†
Lung Contralateral V_5Gy_ (%)	40.7 ± 20.4	37.4 ± 20.2	34.5 ± 19.6	−3.3 ± 4.8	0.006*	−6.2 ± 6.4	<0.001†
Spinal Cord D_0.03 cm3_ (Gy)	38.3 ± 8.6	36.9 ± 8.2	36.1 ± 8.4	−1.4 ± 1.8	0.003*	−2.2 ± 2.2	<0.001†
Skin D_0.03 cm3_ (Gy)	41.6 ± 9.6	40.0 ± 8.9	38.5 ± 8.6	−1.6 ± 2.8	0.021*	−3.1 ± 3.5	<0.001†
Esophagus D_0.03 cm3_ (Gy)	55.9 ± 12.2	54.8 ± 12.3	54.4 ± 12.1	−1.0 ± 2.1	0.039*	−1.5 ± 4.3	0.132
Heart D_0.03 cm3_ (Gy)	58.0 ± 14.9	57.6 ± 16.0	57.4 ± 16.6	−0.4 ± 2.4	0.450	−0.6 ± 3.5	0.427
MedEnv_05 D_0.03 cm3_ (Gy)	64.9 ± 1.0	65.1 ± 1.0	65.3 ± 1.0	0.2 ± 0.5	0.145	0.4 ± 0.6	0.017*
Body D_0.03 cm3_ (Gy)	65.4 ± 1.1	65.5 ± 1.0	65.7 ± 1.0	0.1 ± 0.6	0.462	0.3 ± 0.7	0.077

**B. Combined scenario**	Plan_8 (Reference)	Dose A (Plan_8 + Plan_5)	Dose B (Plan_8 + Plan_2)	Difference Dose A – Plan_8	P-value	Difference Dose B − Plan_8	P-value

Body D_mean_ (Gy)	7.0 ± 2.4	6.8 ± 2.4	6.7 ± 2.4	−0.2 ± 0.1	<0.001†	−0.3 ± 0.2	<0.001†
Heart D_mean_ (Gy)	5.8 ± 3.7	5.6 ± 3.7	5.5 ± 3.7	−0.2 ± 0.2	0.002*	−0.3 ± 0.4	0.002*
Esophagus D_mean_ (Gy)	18.4 ± 9.4	17.9 ± 9.4	17.6 ± 9.4	−0.5 ± 0.6	0.001*	−0.8 ± 0.9	0.001*
Lungs-GTV D_mean_ (Gy)	12.9 ± 4.2	12.6 ± 4.1	12.3 ± 4.1	−0.3 ± 0.2	<0.001†	−0.6 ± 0.4	<0.001†
Lungs-GTV V_5Gy_ (%)	51.2 ± 18.6	50.3 ± 18.6	49.5 ± 18.2	−0.9 ± 1.1	0.002*	−1.7 ± 1.8	<0.001†
Lung Ipsilateral V_5Gy_ (%)	63.9 ± 19.5	63.4 ± 19.6	63.0 ± 19.6	−0.5 ± 0.7	0.004*	−0.9 ± 1.2	0.003*
Lung Contralaterla V_5Gy_ (%)	40.7 ± 20.4	39.4 ± 20.2	38.3 ± 19.7	−1.3 ± 2.0	0.011*	−2.4 ± 2.9	0.002*
Spinal Cord D_0.03 cm3_ (Gy)	38.3 ± 8.6	37.6 ± 8.7	37.1 ± 8.9	−0.8 ± 1.2	0.009*	−1.3 ± 2.0	0.100
Skin D_0.03 cm3_ (Gy)	41.6 ± 9.6	41.0 ± 9.6	40.2 ± 9.5	−0.6 ± 1.4	0.069	−1.4 ± 1.9	0.004*
Esophagus D_0.03 cm3_ (Gy)	55.9 ± 12.2	55.2 ± 12.3	54.8 ± 12.3	−0.7 ± 1.5	0.059	−1.1 ± 2.8	0.104
Heart D_0.03 cm3_ (Gy)	58.0 ± 14.9	58.1 ± 14.9	58.1 ± 14.8	0.0 ± 0.7	0.829	0.0 ± 1.2	0.919
MedEnv_05 D_0.03 cm3_ (Gy)	64.9 ± 1.0	64.9 ± 1.0	65.0 ± 1.1	0.0 ± 0.4	0.893	0.1 ± 0.5	0.500
Body D_0.03 cm3_ (Gy)	65.4 ± 1.1	65.3 ± 1.1	65.4 ± 1.1	−0.1 ± 0.4	0.268	0.0 ± 0.6	0.855

**Fig. 3:**
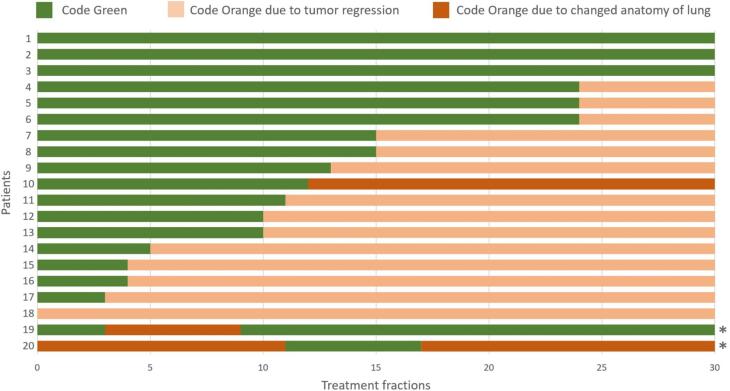
Distribution of code green, orange and red (did not occur) classifications during the 30 fractions of the treatment for all 20 patients. Green: Classified code green due to no/minimal anatomical changes. Light orange: Classified code orange due to tumor regression. Dark orange: Classified code orange due to changed anatomy of lung. An asterisk indicates a treatment plan adaptation.

The results of all CBCT classifications according to the IGRT protocol are depicted in Fig. 3. Of the 600 (20 patients x 30 fractions) treatment fractions, 49% (294/600) were classified as code green and 51% (306/600) as code orange. Three out of 20 patients were classified code green for 30/30 fractions indicating they had no or minimal changes for the entire treatment course. One patient was classified code orange for 30/30 fractions, indicating this patient had anatomical changes from the start of treatment. The other 80% of the patients had a combination of code green classifications at the start of treatment and code orange classifications later on in the treatment. The type of anatomical change that occurred was tumor regression (14/20) and changed anatomy of the lung (3/20), e.g., changes in pleural effusion or atelectasis. Tumor regression was responsible for 84% (258/306) of the code orange classified fractions. Two patients had a plan adaptation which was caused by a changed anatomy of the lung. One of these patients remained code green after adaptation. Table 2B shows the results of all OAR for the combined scenarios (Dose A, or Dose B), combining the 8 mm plans with the 5 mm or 2 mm plans, depending on the classification of the CBCT (code green, code orange). OAR dose was still lower compared to the reference dose, and the maximum dose did not increase.

The CTVp dose coverage on the CBCT for all fractions (code green and orange) and the three treatment plans (Plan_8, Plan_5, and Plan_2) are depicted in Supplementary Fig. S2. Three patients were excluded from this evaluation since their treatment field exceeded the CBCT field of view (FOV) and the CTVp coverage could not be accurately determined. Dose A (combining Plan_8 with Plan_5) resulted in 14 patients with an average CTVp V_95%_ coverage of more than 99% over the course of treatment, compared to 11 patients in Dose B (combining Plan_8 with Plan_2), and 14 patients in Plan_8 (reference).

Table 3 shows a summary of the CTVp coverage per fraction for the treatment fractions that were classified as code green (N = 230). The CTVp V_95%_ coverage of the treatment fractions remained above 95% in 94% of the fractions in the 5 mm plans and 87% for the 2 mm plans, compared to 98% for the reference plans. Coverage of the CTVp was less than 95% in 29 fractions in the 2 mm plans and 13 of the fractions in the 5 mm plans, compared to 4 fractions in the reference plans. The majority of the fractions with a V_95%_ coverage lower than 95 % was caused by 1 patient, i.e. 14 of 29 fractions for Plan_2, 10 of 13 fractions for Plan_5, and 2 of 4 fractions for Plan_8. For the CTV with 1 mm expansion, the portion of fractions where V_95%_ was larger than 95% and 99%, was lowest for Plan_2, with 49% and 10% of the fractions respectively, while in Plan_5 this was 90% and 50%.

**Table 3:** Number of CBCTs with a CTV and CTV + 1m m V95% coverage of more than 90%, 95% and 99%, based on the individual CBCT dose recalculations, for the reference treatment plan (Plan_8) and the two reduced PTV margin plans (Plan_5 and Plan_2). Three patients were excluded since they had a target volume that exceeded the field of view of the CBCT and CTV coverage could not be determined and only code green CBCTs (no or minimal anatomical changes) are taken into account (N = 230). CBCT: Cone-beam CT, CTV: Clinical target volume.

	CTV V_95%_, # CBCTs (%)				CTV + 1 mm V_95%_, # CBCTs (%)		
	≥90%	≥95%	≥99%		≥90%	≥95%	≥99%
Plan_8 (ref)	229 (100)	226 (98)	206 (90)		229 (100)	219 (95)	192 (83)
Plan_5	228 (99)	217 (94)	173 (75)		221 (96)	206 (90)	114 (50)
Plan_2	219 (95)	201 (87)	94 (41)		203 (88)	112 (49)	24 (10)

## 4 Discussion

In this study we investigated to what extent reduced margins could lead to lower OAR doses, and if it was feasible to implement this in the clinic using an IGRT protocol for ART. Patients with no or minimal anatomical changes on CBCT during the entire treatment benefitted the most from reduced margin plans. However, it is not known in advance which patients will develop anatomical changes during treatment and when these changes will occur. Selection of patients using the IGRT protocol for treatment with reduced margin plans proved feasible, and led to decreased OAR dose for all patients.

Our previous study analyzed an IGRT protocol for ART in lung cancer [12] and showed that in 21% of the patients no anatomical changes were observed during treatment. Additionally, in 57% of patients an anatomical change was observed in only one to three fractions during treatment. This indicates that a large portion of patients could benefit from treatment plans with a smaller PTV margin. In the present study, 49% of the treatment fractions showed no or minimal anatomical changes and were assigned a reduced margin treatment plan, and three patients (15%) showed no or minimal changes for their entire treatment.

Tumor regression is a very common anatomical change that occurs over the course of treatment [23]. In this study, 14 of 20 patients had treatment fractions that were classified as code orange due to tumor regression, and were therefore not eligible for treatment with smaller PTV margin plans. Per fraction dose recalculations on the CBCTs in patients with tumor regression showed a CTV coverage >95% in 90% of Plan_5 treated fractions and 81% of Plan_2 treated fractions. The fractions that had a coverage <95% were mainly caused by two patients with a systematically lower coverage of the CTV. It could be investigated if a smaller PTV margin can be safely used in fractions with tumor regression, e.g., possibly in the subgroup tumor regression without shift, to further increase the number of fractions that are assigned the reduced PTV margin plans.

We observed modest improvements in OAR doses when reducing the PTV margin from 8 mm to 5 or 2 mm. The benefits may be limited due to the primary tumor being located in the lungs, at some distance to the heart and the esophagus, which are located in the mediastinum. The OAR dose is also dependent on the location and extent of the nodal tumor volume. The involved nodes are located in the mediastinum and/or the hilus, but its margin is not reduced in this study. Additionally, even though the improvement was relatively small for the entire study cohort in the combined scenario, reduction of OAR doses was possible in patients that had a majority of fractions with no or minimal anatomical changes (Table 2).

In this study the coverage of the CTV was >95% in most of the fractions treated with the 5 mm or 2 mm PTV margin plans (94% or 87%, respectively). However, for the CTV expanded with 1 mm, the coverage was >95% in 90% for Plan_5 and decreased to 49% for Plan_2, indicating a 2 mm PTV margin is potentially insufficient. Møller et al. investigated an ART protocol to ensure target coverage and decrease dose to the lungs [24]. Treatments were adapted when the CTV V_95%_ coverage dropped by more than 1% with respect to the reference plan. If this rule was applied to our cohort, then 187/230 (81%) code green fractions were eligible for the 5 mm PTV margin plan, and 100/230 (43%) fractions for the 2 mm PTV margin plan. In our study the majority of fractions with a coverage below 95% originated from one patient. In this patient the reference plan also showed a decreased coverage over the treatment course. This was the only patient with a systematic shift of the tumor outside the CTV. In the IGRT protocol this criterium is currently classified as a code green, since it is not relevant for necessitating a plan adaptation [12], but for safe PTV margin reduction it might be relevant and should be investigated further.

Few other studies have evaluated the possibilities of reducing PTV margins in lung cancer. The study of van Diessen et al. [25] showed a significant reduction of grade 3 dysphagia and pulmonary toxicity when the PTV margin was reduced with 2–3 mm for both the primary tumor and lymph nodes, however the total dose to the PTV was also decreased from 70 Gy (EQD2) in the reference cohort to 60 Gy in the reduced margin cohort. The study of Nelson et al. [26] investigated the possibilities of reduced PTV margins for dose escalation to the tumor and found that PTV dose could be increased in the majority of patients. Both studies investigated a general reduction of margins, but this could lead to an increase of the number of patients that require a plan adaptation during treatment. By using an IGRT protocol to identify patients that have no or minimal anatomical changes, a reduced margin plan can be safely administered to eligible patients. As far as we know there are no other studies that investigated a library of plans or plan of the day approach for ART in lung cancer to reduce PTV margins and improve OAR dose in patients with no or minimal anatomical changes.

This study has several limitations. First, we only considered PTV margin reductions to 5 mm and 2 mm. Our study setup takes into account some uncertainties (setup, daily anatomical variation, intra/inter-fraction motion, delineation), but omits other uncertainties (e.g., machine delivery uncertainties, kV-MV isocenter agreement), making a 2 mm margin unrealistic to implement in clinical practice. There may be different PTV margin reductions that present a more optimal tradeoff between OAR dose reduction while retaining CTV coverage. Evaluating the full range of margin reductions could prove insightful. Since this would be quite labor intensive, automated treatment planning approaches could make this type of evaluation more manageable.

Second, the effect on OAR dose was evaluated on the planning CT, since it includes delineated OAR structures which are not always completely available for evaluation on the CBCT due to the limited FOV. Evaluation on the planning CT makes the evaluation of different dose parameters, e.g., mean dose, total dose, possible and provides an interpretable estimation of the dose to the OAR in the adjusted margin scenarios. This method is often used in dose comparison studies, but does not consider daily anatomical variations of the OAR. Synthetic CTs generated from daily CBCTs, extended with planning CT information outside the CBCT FOV, might be an option to determine the OAR dose including their daily variations [27], [28], [29]. However, the location of OAR outside the CBCT FOV are assumed based on planning CT information, potentially limiting the accuracy of the estimated OAR dose.

Third, for the CTV coverage evaluation a CBCT dose recalculation was performed using the actual online rigid registrations between all CBCTs and the planning CT. In 17/20 patients the CTV and treatment fields were within the CBCT FOV. Dose accumulation of the CBCT dose recalculations could give more insight into the overall treatment dose [30]. However, dose accumulation requires deformable image registration and dose warping, which introduces new uncertainties to the evaluation and this makes interpretation more difficult compared to a direct evaluation on the CBCT.

In conclusion, a plan of the day approach for PTV margin reduction in NSCLC patients was presented that lowered OAR doses while maintaining CTV coverage. The IGRT protocol was able to select eligible fractions for treatment with smaller margins when there were no or minimal anatomical changes present. We investigated two scenarios of different PTV margin reductions, where 5 mm was preferred to ensure adequate CTV coverage.

## Declaration of competing interest

The authors declare that they have no known competing financial interests or personal relationships that could have appeared to influence the work reported in this paper.
