# Supplementary material for: Adaptive treatment margins to reduce organs at risk dose in patients with no or minimal anatomical changes in radiotherapy of non-small cell lung cancer
Source: Phys Imaging Radiat Oncol. 2025 Jan 20;33:100699. doi: 10.1016/j.phro.2025.100699 (PMC11810835; doi:10.1016/j.phro.2025.100699)
Supplement: Supplementary Data 1 [file mmc1.pdf]

Supplementary Materials

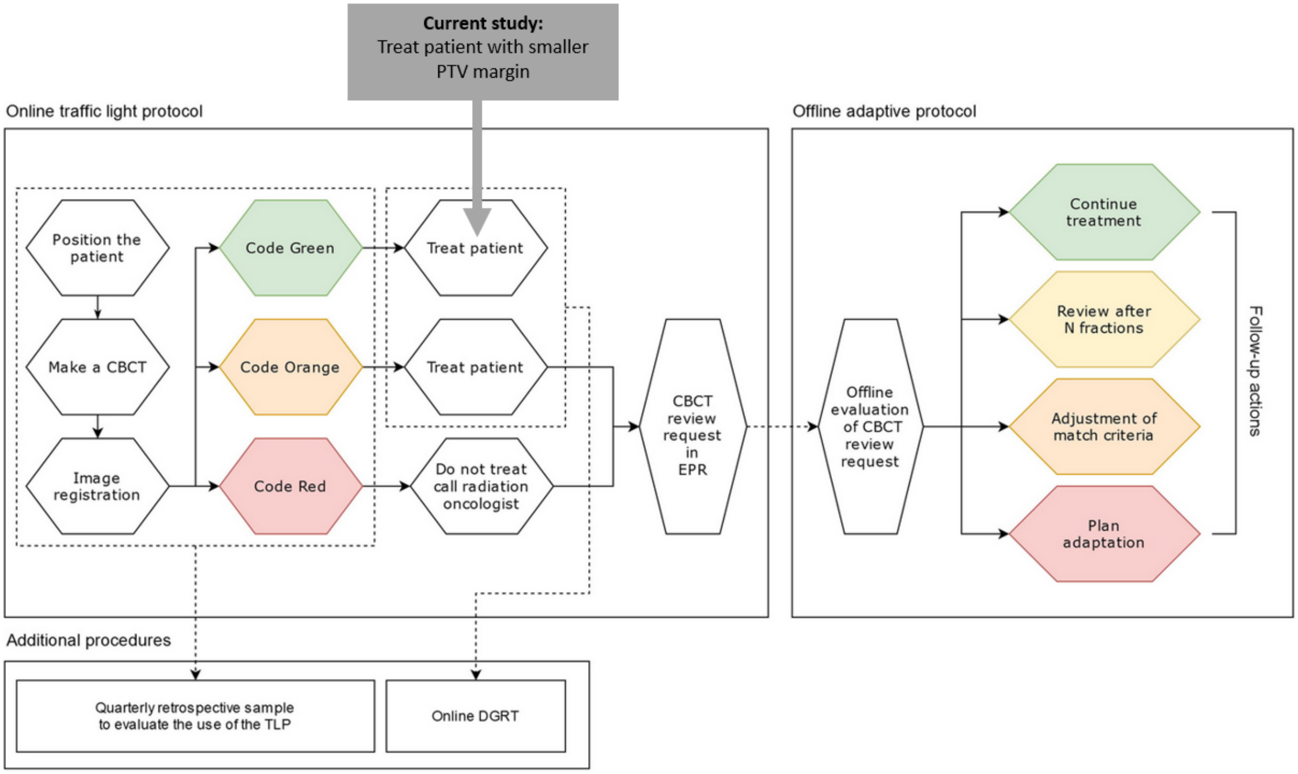

Supplementary Figure S1: The IGRT protocol used for position verification and identifying patients for treatment plan adaptation. In our study the CBCTs that are classified code green are assigned reduced PTV margin plans in this study. IGRT: Image-guided radiotherapy, CBCT: Cone-beam CT, PTV: Planning target volume, EPR: Electronic patient record, TLP: Traffic light protocol, DGRT: Dose-guided radiotherapy.

Supplementary Table S1: Criteria of the three classifications in the online IGRT protocol. Selection is based on visual evaluation by the dosimetrist at the treatment machine. IGRT: Image-guided radiotherapy, CBCT: Cone-beam CT, CTV: Clinical target volume, PRV: Planning risk volume, PTV: Planning target volume.

Classification	Criteria to evaluate on the CBCT
Code Green	Deviation of bony anatomy of less than 5 mm around the target volume, the visible tumor is within the CTV, no change of anatomy of the lung (e.g., no change in atelectasis or pleural effusion), no change in tumor volume, mediastinum (near tumor/nodes) inside PRV (i.e., mediastinum expanded by 5 mm). Depending on registration method: carina inside PRV (carina expanded by 2 mm), critical isodose structure (54 Gy) outside spinal cord.
Code Orange	Deviation of bony anatomy of more than 5 mm around the target volume. Partly changed anatomy of lung (e.g., atelectasis or pleural effusion), changed tumor volume, shift of tumor outside CTV but inside PTV, shift of mediastinum (near tumor/nodes) outside the PRV (mediastinum +5 mm). Depending on registration method: carina outside PRV (carina +2 mm).
Code Red	Shift of tumor outside the PTV, tumor not visible, complete regression, complete change of anatomy of the lungs (e.g., atelectasis, pleural effusion). Depending on registration method: critical isodose structure (54 Gy) inside spinal cord.

pat id	CTVp V95% (%) per fraction	CB1	CB2	CB3	CB4	CB5	CB6	CB7	CB8	CB9	CB10	CB11	CB12	CB13	CB14	CB15	CB16	CB17	CB18	CB19	CB20	CB21	CB22	CB23	CB24	CB25	CB26	CB27	CB28	CB29	CB30	Average
	Plan_2	95	96	97	98	96	99	99	97	98	96	97	98	97	99	98	95	93	97	95	96	98	84	99	96	99	96	97	96	98	99	97
	Plan_5	99	99	99	100	99	100	100	99	100	99	100	100	100	100	100	98	98	100	99	99	100	86	100	99	100	99	100	99	100	100	99
1	Plan_8	100	100	100	100	99	100	99	99	99	99	99	100	99	100	100	100	100	100	100	100	100	88	100	100	100	100	100	100	100	100	99
	Dose A	99	99	99	100	99	100	100	99	100	99	100	100	100	100	100	98	98	100	99	99	100	86	100	99	100	99	100	99	100	100	99
	Dose B	95	96	97	98	96	99	99	97	98	96	97	98	97	99	98	95	93	97	95	96	98	84	99	96	99	96	97	96	98	99	97
	Plan_02	100	96	97	96	95	96	95	95	91	95	99	99	100	99	97	99	100	100	98	100	100	100	100	100	100	100	100	100	100	100	98
	Plan_05	100	98	99	99	98	98	98	98	96	98	100	100	100	100	100	100	100	100	100	100	100	100	100	100	100	100	100	100	100	100	99
2	Plan_08	100	100	100	100	100	100	100	100	100	100	100	100	100	100	100	100	100	100	100	100	100	100	100	100	100	100	100	100	100	100	100
	Dose A	100	98	99	99	98	98	98	98	96	98	100	100	100	100	100	100	100	100	100	100	100	100	100	100	100	100	100	100	100	100	99
	Dose B	100	96	97	96	95	96	95	95	91	95	99	99	100	99	97	99	100	100	98	100	100	100	100	100	100	100	100	100	100	100	98
3																																
	Plan_02	100	100	100	100	99	100	100	100	100	100	100	100	100	100	100	100	100	100	100	100	100	100	100	100	100	100	100	100	100	100	100
	Plan_05	100	100	100	100	100	100	100	100	100	100	100	100	100	100	100	100	100	100	100	100	100	100	100	100	100	100	100	100	100	100	100
4	Plan_08	100	100	100	100	100	100	100	100	100	100	100	100	100	100	100	100	100	100	100	100	100	100	100	100	100	100	100	100	100	100	100
	Dose A	100	100	100	100	100	100	100	100	100	100	100	100	100	100	100	100	100	100	100	100	100	100	100	100	100	100	100	100	100	100	100
	Dose B	100	100	100	100	99	100	100	100	100	100	100	100	100	100	100	100	100	100	100	100	100	100	100	100	100	100	100	100	100	100	100
	Plan_02	99	98	99	99	100	100	100	99	100	100	100	100	100	100	99	100	100	100	100	100	100	100	100	100	100	100	100	100	99	100	100
	Plan_05	100	100	100	100	100	100	100	100	100	100	100	100	100	100	100	100	100	100	100	100	100	100	100	100	100	100	100	100	100	100	100
5	Plan_08	100	100	100	100	100	100	100	100	100	100	100	100	100	100	100	100	100	100	100	100	100	100	100	100	100	100	100	100	100	100	100
	Dose A	100	100	100	100	100	100	100	100	100	100	100	100	100	100	100	100	100	100	100	100	100	100	100	100	100	100	100	100	100	100	100
	Dose B	99	98	99	99	100	100	100	99	100	100	100	100	100	100	99	100	100	100	100	100	100	100	100	100	100	100	100	100	100	100	100
6																																
	Plan_02	97	97	100	89	97	98	98	96	99	97	99	99	99	100	99	97	100	99	96	98	99	99	98	100	98	98	97	99	100	99	98
	Plan_05	99	98	100	91	98	99	99	98	100	99	100	100	100	100	100	98	100	100	99	100	100	100	99	100	100	99	99	100	100	100	99
7	Plan_08	100	99	100	96	100	100	100	99	100	99	100	100	100	100	100	99	100	100	100	100	100	100	100	100	100	100	100	100	100	100	100
	Dose A	99	98	100	91	98	99	99	98	100	99	100	100	100	100	100	99	100	100	100	100	100	100	100	100	100	100	100	100	100	100	99
	Dose B	97	97	100	89	97	98	98	96	99	97	99	99	99	100	99	99	100	100	100	100	100	100	100	100	100	100	100	100	100	100	99
	Plan_02	92	95	92	97	93	86	89	89	89	81	88	89	87	92	86	86	91	83	91	81	91	87	88	90	85	86	91	88	93	90	89
	Plan_05	96	97	97	98	97	91	94	91	95	86	94	94	92	94	92	90	95	89	93	86	94	93	93	93	91	92	94	94	97	94	93
8	Plan_08	98	99	99	99	98	95	97	95	98	93	98	97	96	96	96	94	97	94	96	93	97	98	96	97	95	96	97	97	99	97	97
	Dose A	96	97	97	98	97	91	94	91	95	86	94	94	92	94	92	94	97	94	96	93	97	98	96	97	95	96	97	97	99	97	95
	Dose B	92	95	92	97	93	86	89	89	89	81	88	89	87	92	86	94	97	94	96	93	97	98	96	97	95	96	97	97	99	97	93
	Plan_02	100	99	98	99	98	97	97	94	97	97	99	97	96	98	92	100	100	100	100	99	94	95	92	91	95	89	93	97	94	100	97
	Plan_05	100	100	99	100	100	100	100	97	100	99	100	100	99	100	98	100	100	100	100	100	98	98	97	96	98	94	96	100	98	100	99
9	Plan_08	100	100	100	100	100	100	100	99	100	100	100	100	100	100	100	100	100	100	100	100	100	100	99	99	100	98	99	100	100	100	100
	Dose A	100	100	99	100	100	100	100	97	100	99	100	100	99	100	100	100	100	100	100	100	100	100	99	99	100	98	99	100	100	100	100
	Dose B	100	99	98	99	98	97	97	94	97	97	99	97	96	100	100	100	100	100	100	100	100	100	99	99	100	98	99	100	100	100	99
	Plan_02	99	98	96	100	97	98	96	98	97	98	98	98	97	96	94	99	98	99	99	99	98	100	100	100	100	100	100	100	100	100	98
	Plan_05	100	99	98	100	99	99	99	100	99	100	100	100	99	98	97	100	99	100	100	100	99	100	100	100	100	100	100	100	100	100	99
10	Plan_08	100	100	99	100	100	100	99	100	100	100	100	100	100	99	97	100	99	100	100	100	99	100	100	100	100	100	100	100	100	100	100
	Dose A	100	99	98	100	99	99	99	100	99	100	100	100	100	99	97	100	99	100	100	100	99	100	100	100	100	100	100	100	100	100	100
	Dose B	99	98	96	100	97	98	96	98	97	98	98	98	100	99	97	100	99	100	100	100	99	100	100	100	100	100	100	100	100	100	99
	Plan_02	98	98	98	98	97	96	98	98	97	96	97	97	98	98	96	95	95	94	97	97	95	97	95	96	96	98	94	96	88	93	96
	Plan_05	100	99	99	100	99	99	99	99	98	98	99	99	99	100	99	97	97	98	99	99	97	99	98	98	98	100	97	98	94	96	98
11	Plan_08	100	100	100	100	100	100	100	100	100	100	100	100	100	100	100	99	99	100	100	100	99	100	100	99	100	100	99	100	98	99	100
	Dose A	100	99	99	100	99	99	99	99	98	98	99	100	100	100	100	99	99	100	100	100	99	100	100	99	100	100	99	100	98	99	99
	Dose B	98	98	98	98	97	96	98	98	97	96	97	100	100	100	100	99	99	100	100	100	99	100	100	99	100	100	99	100	98	99	99
	Plan_02	100	99	99	99	99	100	99	99	100	100	99	99	100	99	100	100	99	99	99	98	99	99	98	99	99	99	99	99	99	99	99
	Plan_05	100	100	100	100	100	100	100	100	100	100	100	100	100	100	100	100	100	100	100	100	100	100	100	100	100	100	100	100	100	100	100
12	Plan_08	100	100	100	100	100	100	100	100	100	100	100	100	100	100	100	100	100	100	100	100	100	100	100	100	100	100	100	100	100	100	100
	Dose A	100	100	100	100	100	100	100	100	100	100	100	100	100	100	100	100	100	100	100	100	100	100	100	100	100	100	100	100	100	100	100
	Dose B	100	99	99	99	99	100	99	99	100	100	100	100	100	100	100	100	100	100	100	100	100	100	100	100	100	100	100	100	100	100	100
13																																
	Plan_02	100	100	99	99	100	100	100	99	99	99	100	100	100	100	100	99	100	100	100	100	98	98	99	96	98	96	99	100	99	99	99
	Plan_05	100	100	100	100	100	100	100	100	100	100	100	100	100	100	100	100	100	100	100	100	99	99	99	97	98	96	99	100	100	100	100
14	Plan_08	100	100	100	100	100	100	100	100	100	100	100	100	100	100	100	100	100	100	100	100	98	98	100	98	99	98	100	100	100	100	100
	Dose A	100	100	100	100	100	100	100	100	100	100	100	100	100	100	100	100	100	100	100	100	98	98	100	98	99	98	100	100	100	100	100
	Dose B	100	100	99	99	100	100	100	100	100	100	100	100	100	100	100	100	100	100	100	100	98	98	100	98	99	98	100	100	100	100	100
	Plan_02	98	97	100	99	99	99	98	99	98	100	98	99	94	99	100	94	100	100	92	93	100	92	96	98	98	99	100	93	100	98	98
	Plan_05	100	99	100	100	100	100	99	100	100	100	99	100	98	100	100	98	100	100	98	98	100	97	99	99	99	100	100	98	100	100	99
15	Plan_08	100	100	100	100	100	100	100	100	100	100	100	100	99	100	100	99	100	100	100	100	100	99	100	100	100	100	100	100	100	100	100
	Dose A	100	99	100	100	100	100	100	100	100	100	100	100	99	100	100	99	100	100	100	100	100	99	100	100	100	100	100	100	100	100	100
	Dose B	98	97	100	99	100	100	100	100	100	100	100	100	99	100	100	99	100	100	100	100	100	99	100	100	100	100	100	100	100	100	100
	Plan_02	98	97	97	98	98	97	99	98	98	98	98	98	98	99	99	99	99	99	99	99	99	98	99	98	99	99	98	99	99	97	98
	Plan_05	99	98	98	98	98	98	99	98	99	99	98	98	99	99	99	99	99	99	99	99	99	99	99	98	99	99	99	99	99	98	99
16	Plan_08	99	99	99	99	99	98	99	99	99	99	99	99	99	99	99	100	99	100	99	99	99	99	99	99	100	100	99	100	100	98	99
	Dose A	99	98	98	98	99	98	99	99	99	99	99	99	99	99	99	100	99	100	99	99	99	99	99	99	100	100	99	100	100	98	99
	Dose B	98	97	97	98	99	98	99	99	99	99	99	99	99	99	99	100	99	100	99	99	99	99	99	99	100	100	99	100	100	98	99
	Plan_02	97	94	95	93	98	91	91	90	88	90	85	93	95	91	99	96	97	99	99	100	99	97	98	97	96	95	99	99	99	99	95
	Plan_05	97	94	96	95	98	94	94	91	88	93	90	96	97	93	100	97	97	99	100	100	99	98	98	98	97	97	100	99	99	100	96
17	Plan_08	97	94	96	95	98	94	94	91	87	93	92	95	96	93	100	97	97	99	100	100	100	99	98	99	98	97	100	99	100	100	97
	Dose A	97	94	96	95	98	94	94	91	87	93	92	95	96	93	100	97	97	99	100	100	100	99	98	99	98	97	100	99	100	100	97
	Dose B	97	94	95	95	98	94	94	91	87	93	92	95	96	93	100	97	97	99	100	100	100	99	98	99	98	97	100	99	100	100	96
	Plan_02	98	99	99	99	99	99	99	100	99	99	99	99	98	99	99	99	99	99	99	99	100	99	99	99	99	100	100	100	99	99	99
	Plan_05	100	100	100	100	100	100	100	100	100	100	100	100	100	100	100	100	100	100	100	100	100	100	100	100	100	100	100	100	100	100	100
18	Plan_08	100	100	100	100	100	100	100	100	100	100	100	100	99	100	100	100	100	100	100	100	100	100	100	100	100	100	100	100	100	100	100
	Dose A	100	100	100	100	100	100	100	100	100	100	100	100	99	100	100	100	100	100	100	100	100	100	100	100	100	100	100	100	100	100	100
	Dose B	100	100	100	100	100	100	100	100	100	100	100	100	99	100	100	100	100	100	100	100	100	100	100	100	100	100	100	100	100	100	100
	Plan_02/Plan_02b	99	99	95	99	98	99	91	96	95	97	100	93	97	94	97	99	97	97	91	96	96	95	91	92	100	98	96	100	97	97	96
	Plan_05/Plan_05b	100	100	99	100	100	100	96	99	99	99	100	98	100	98	100	100	100	100	97	99	99	99	96	98	100	100	99	100	99	100	99
19	Plan_08/Plan_08b	100	99	99	100	99	100	100	100	100	100	100	100	100	100	100	100	100	100	99	100	100	100	99	100	100	100	100	100	100	100	100
	Dose A	100	100	99	100	99	100	100	100	100	99	100	98	100	98	100	100	100	100	97	99	99	99	96	98	100	100	99	100	99	100	99
	Dose B	99	99	95	100	99	100	100	100	100	97	100	93	97	94	97	99	97	97	91	96	96	95	91	92	100	98	96	100	97	97	97
	Plan_02/Plan_02b	90	82	81	82	89	69	74	75	72	75	72	100	100	99	100	100	98	98	99	90	99	92	99	98	99	99	100	100	99	100	91
	Plan_05/Plan_05b	97	91	90	92	97	79	84	83	78	81	77	100	100	100	100	100	98	99	100	91	100	95	100	99	99	100	100	100	100	100	94
20	Plan_08/Plan_08b	98	94	93	93	97	83	87	85	78	82	77	100	100	100	100	100	99	99	100	93	99	98	100	99	99	99	100	100	99	100	95
	Dose A	98	94	93	93	97	83	87	85	78	82	77	100	100	100	100	100	98	99	100	93	99	98	100	99	99	99	100	100	99	100	95
	Dose B	98	94	93	93	97	83	87	85	78	82	77	100	100	99	100	100	98	99	100	93	99	98	100	99	99	99	100	100	99	100	95

Supplementary Figure S2: CBCT dose recalculations showing the CTV dose per fraction for all 30 fractions and for all three treatment plans and combined doses A and B. Dark green indicates a CTV coverage >99%, light green indicates a CTV coverage >95%, light orange indicates a CTV coverage <95% and dark orange indicates a CTV coverage <90%. Red vertical bars indicate a change in IGRT protocol classification from code green to code orange. Black vertical bars indicate a plan adaptation. The grey horizontal bars indicate the three patients that were excluded from this evaluation due to the tumor size/treatment field that exceeded the field of view of the CBCT. CBCT: cone-beam CT, CTV: Clinical target volume, IGRT: image-guided radiotherapy.
